# Profiles of paediatric patients experiencing stroke-like episodes associated with mitochondrial disease

**DOI:** 10.3389/fneur.2025.1657852

**Published:** 2025-12-08

**Authors:** Gülhan Karakaya Molla, Özlem Ünal Uzun, Hanım Babazade Agakisili, Emine Genç, Zümrüt Arslan Gülten, Tarık Yıldırım, Aydan Sezgin Ersoy, Belkıs Ak, Aliye Gülbahçe, Sevil Yıldız, Nafiye Emel Çakar, Meryem Karaca, Tanyel Zübarioğlu, Burcu Öztürk Hişmi, Şahin Erdöl, Hasan Önal, Bülent Kara, Gülden Fatma Gökçay

**Affiliations:** 1 Department Child Health and Diseases Child Metabolism, Tekirdağ İsmail Fehmi Cumalıoğlu City Hospital, Tekirdağ, Türkiye; 2 Department Child Health and Diseases Child Metabolism, Kocaeli University Faculty of Medicine, İzmit, Türkiye; 3 Department Child Health and Diseases Child Metabolism, Istanbul University Cerrahpaşa Faculty of Medicine, Fatih, Türkiye; 4 Department Child Health and Diseases Child Metabolism, Marmara University Faculty of Medicine, Istanbul, Türkiye; 5 Department Child Health and Diseases Child Metabolism, Şişli Hamidiye Etfal Training and Research Hospital, Şişli, Türkiye; 6 Department Child Health and Diseases Child Metabolism, Istanbul Başakşehir Pine and Sakura City Hospital, Başakşehir, Türkiye; 7 Department Child Health and Diseases Child Metabolism, Uludağ University Faculty of Medicine, Bursa, Türkiye; 8 Department Child Health and Diseases Child Metabolism, Istanbul University Istanbul Faculty of Medicine, Istanbul, Türkiye; 9 Department Child Health and Diseases Child Metabolism, Kocaeli City Hospital, İzmit, Türkiye; 10 Department Child Health and Diseases Child Metabolism, Bursa High Specialized Hospital Children's Hospital, Bursa, Türkiye; 11 Department Child Health and Diseases Child Metabolism, Istanbul Prof. Dr. Cemil Taşcıoğlu City Hospital, Istanbul, Türkiye; 12 Department Child Health and Diseases Child Neurology, Kocaeli University Faculty of Medicine, İzmit, Türkiye

**Keywords:** stroke-like episodes, mitochondrial diseases, MELAS, POLG mutations, CoQ10 deficiency

## Abstract

**Introduction:**

Stroke-like episodes (SLE) are defined as events characterized by the sudden onset of neurological symptoms with clinical manifestations similar to those of a stroke. However, they are distinguished by the presence of radiological lesions that do not conform to single vascular territory. MELAS syndrome, which is characterized by metabolic encephalopathy, lactic acidosis, and SLE, has been identified as the first genetically defined and most widely known mitochondrial cause of SLE. It has been demonstrated that SLE may occur in the course of a variety of mitochondrial diseases, including those that are the result of nuclear DNA mutations.

**Objective:**

In this retrospective, multicenter, observational cohort study, we sought to determine the clinical, radiological, EEG, and genetic characteristics of patients with mitochondrial gene mutations presenting with SLE and the frequency and treatment of SLE.

**Methods:**

Thirty-four patients with a genetically diagnosed mitochondrial disease from 9 paediatric metabolic disease centres in the Marmara Region of Turkey were included in the study, of whom 13 pateints had SLEs. Demographic characteristics, symptoms, clinical features, cranial MRI, EEG findings, and genetic characteristics were evaluated.

**Conclusion:**

In this study, stroke-like episodes in genetically defined mitochondrial disorders were most frequently observed in MELAS and POLG mutations, and rarely in CoQ10 deficiency, Leigh syndrome cases. Cranial MRI findings are often frontotemporal in location and inconsistent with vascular distribution, and focal epileptiform activity on EEG are diagnostically significant. In MELAS, clinical improvement was observed in patients when L-arginine was initiated in the acute period. The findings emphasise that SLE should be evaluated in the differential diagnosis of sudden onset neurological symptoms in mitochondrial diseases.

## Introduction

Stroke is an important neurologic emergency that may lead to serious morbidity and mortality, although it is observed more rarely in childhood than in adults. The World Health Organization defines stroke as “a rapidly developing set of clinical findings due to focal or global impairment of cerebral functions, lasting longer than 24 h or resulting in death” (1). While the etiologies of stroke in the pediatric population spread over a wide spectrum, hereditary metabolic diseases are among the rarer causes. Nevertheless, they have special importance in terms of the need for early diagnosis and specific treatment (2, 3). SLE are characterized by acute or subacute onset neurologic findings do not conform to single vascular territory (4). The best-defined example in the mitochondrial disease group with SLE is MELAS (mitochondrial encephalomyopathy, lactic acidosis, and stroke-like episodes) syndrome (5). In addition, Kearns-Sayre syndrome, Leigh syndrome, MERRF, LHON (Leber’s hereditary optic neuropathy), and non-syndromic mitochondrial diseases, especially *POLG* mutations, have also been associated with similar clinical pictures (6, 7). The clinical spectrum is quite broad. While migraine-like headaches and epileptic seizures are frequently seen before the episode, various symptoms, including altered consciousness, hemiparesis, aphasia, cortical visual loss, and neuropsychiatric findings, may occur in the acute phase of the episode (7, 8). On imaging, lesions are frequently localized in occipital, parietal, or posterior temporal lobes, limited to the cortex, asymmetric, and incompatible with vascular distribution (4, 9). Electroencephalography (EEG) findings contribute to the diagnosis of encephalopathic changes and focal epileptiform activity (10). Treatment aims to reduce metabolic stress, ensure seizure control, and avoid agents with the potential for mitochondrial toxicity. L-arginine treatment administered in the acute period has been reported to provide symptomatic improvement in some patients (11, 12).

In this study, we retrospectively evaluated the clinical, laboratory, imaging, and EEG characteristics of SLE in children with molecularly confirmed mitochondrial disease who were followed up in nine different pediatric metabolic centers across Turkey.

## Methods

The retrospective medical records of 34 patients who were followed up in the pediatric metabolism and nutrition divisions of nine centers in the Marmara Region in Turkey were analyzed. Inclusion criteria were children and young adults with a genetically confirmed mitochondrial disease and at least one follow-up visit in the participating centers. Exclusion criteria were incomplete molecular diagnosis or lack of accessible clinical data. Current age, age at first episode and age at diagnosis, clinical features, growth parameters, laboratory, radiological, EEG and molecular analysis findings were recorded. For cases with incomplete EEG or MRI data, the available findings were included and explicitly noted as missing when absent. To ensure comparability, standardized data collection templates were used across all nine centers, and case information was harmonized by a coordinating investigator. The data obtained in the study were analyzed using SPSS 23 (Statistical Package for Social Sciences) software. In statistical analyses, Chi-square analysis and Fisher Exact test were used to examine the relationships between categorical variables. Kruskal-Wallis test and Mann- Whitney U test were used to evaluate the differences between continuous variables since parametric test assumptions were not met. In all analyses, the significance level was determined as *p* < 0.05 and the confidence level was evaluated as 95%. Ethical approval was obtained from the Tekirdağ Dr. İsmail Fehmi Cumalıoğlu City Hospital Clinical Research Ethics Committee (Decision no. 2024/119). Research was conducted accordance with the Declaration of Helsinki.

## Findings

### Demographic characteristics

Among the 34 patients with a genetic diagnosis of mitochondrial diseases, 50% (*n* = 17) were female and 50% (*n* = 17) were male. Regarding age distribution, the highest proportion was observed in the 5.01–10-year age group at 23.5% (*n* = 8), whereas the lowest proportion was in the 10.01–15-year age group at 14.7% (*n* = 5). The most common period for the onset of initial symptoms was under 5 years of age, accounting for 58.8% (*n* = 20), while the lowest frequency was recorded in the 15.01–20-year age group at 3% (*n* = 1). In terms of age at diagnosis, 41.2% (*n* = 14) of the patients were diagnosed before the age of 5 years, whereas 14.7% (*n* = 5) were diagnosed after the age of 20 years (Tables 1, 2).

**Table 1:** Demographic characteristics variables are presented as frequency (n) and percentage (%).

Variable	Category	*n*	%
Gender	Male	17	50
	Female	17	50
Age (year)	<5	7	20.6
	5.01–10	8	23.5
	10.01–15	5	14.7
	15.01–20	8	23.5
	Over 20	6	17.7
Age at onset of first symptoms (year)	< 5	20	58.8
	5.01–15	6	17.6
	10.01–15	3	8.8
	15.01–20	1	3
	Over 20	4	11.8
Age at diagnosis (year)	<5	14	41.2
	5.01–10	5	14.7
	10.01–15	8	23.5
	15.01–20	2	5.9
	Over 20	5	14.7
Duration of follow-up (year)	<1	11	32.3
	1.01–2	6	17.6
	2.01–3	5	14.7
	3.01–4	5	14.7
	>4	7	20.5
Diagnosis	MELAS	15	44.1
	Coenzyme Q10 deficiency	9	26.5
	Leigh syndrome	4	11.8
	POLG	3	8.8
	FBXL	2	5.9
	LHON	1	2.9

MELAS, Mitochondrial encephalomyopathy, lactic acidosis, and stroke-like episodes syndrome; LHON, Leber’s hereditary optic neuropathy.

**Table 2:** Clinical findings of the patients.

Patient No	Gender	Age (years)	Diagnosis	Mutation	Age at onset of first symptoms (months)	Age at diagnosis (months)	Number of stroke-like episodes	Localization of lesions on cranial MRI	EEG findings	Lactate levels mmol/L (last viewed)	Duration of Arginine use
1	Female	40	MELAS	*MT-TK*-M8362T	444	480		No lesion		21	2 months
2	Female	28	MELAS	*TRLN1*-A3243G	300	324		No lesion	Frontal deceleration	2.5	22 days
3	Male	16,9	MELAS	*TRLN1*-A323G	18	60		Frontal		34	3 months
4	Male	9	MELAS	*MT-TK*-M3290T > C	12	12	2	Parietal	Epileptiform activity in the left fronto-temporal region	40	
5	Female	50	MELAS	*TRLN1*-A3243G	444	564		Frontal			22 days
6	Male	2	MELAS	*MT-TW*- 5543 T > C	34	36		No lesion		3.2	
7	Male	11,1	MELAS	*MT-TK*-M8362T	84	132		No lesion		44	3 months
8	Male	40	MELAS	*TRLN1*-Nt3243	432	468	4	Parietal	Epileptiform activity in the left fronto-temporal region	7.4	
9	Female	5	MELAS	*MT-TW-*5543 T > C	12	12		No lesion		4	
10	Female	3,7	MELAS	*TRLN1*-m3243A > G	5	36		No lesion	Temporo parietal bilateral epileptiform activity		9 days
11	Male	9	MELAS	*MT-TL1* m3271T > C	88	96	2	Occipital	Low background activity and rare spike-like waves in the right parieto-occipital		11 days
12	Female	15,3	MELAS	*TRLN1*-A3243G	84	156	2	No lesion	Left hemisphere epileptiform activity	7	40 days
13	Male	5,8	MELAS	*TRLN1*-A323G	6	36	1	Temporal		67	11 months
14	Male	18,9	MELAS	*TRLN1*-A323G	144	144		Frontal		3.9	
15	Male	18	MELAS	*MT-TL1* m3271T > C	156	180	5	Parietal		60	34 days
16	Female	21,8	Coenzyme Q10 Deficiency	*COQ8A* c.911C > T hom	204	216		No lesion	Disorganization in the left fronto-temporal region	3.1	
17	Male	4,5	Coenzyme Q10 Deficiency	*COQ4* c.437 T > G	2,5	12	2	Temporal		5.9	
18	Female	17,8	Coenzyme Q10 Deficiency	*COQ8* c.811C > T (p.ARG)	18	180		No lesion	Multifocal generalized epileptiform anomaly	1.4	
19	Female	19,1	Coenzyme Q10 Deficiency	*COQ8A* c.914A > T hom	20	30	1	Frontal		3.6	1 day
20	Male	22,9	Coenzyme Q10 Deficiency	*COQ8B* hom. c1199dup	96	252		No lesion		5.5	
21	Male	13	Coenzyme Q10 Deficiency	*COQ8A* c.811 C > T (p.ARG)	48	144		No lesion	Multifocal generalized epileptiform anomaly	7	
22	Female	6,6	Coenzyme Q10 Deficiency	*COQ8A* c.1344_1345dup hom.	48	60		No lesion		1.49	
23	Male	6,00	Coenzyme Q10 Deficiency	*COQ4* c.437 T > G (p.Phe146C)	48	48		No lesion		3.50	
24	Female	10,9	Coenzyme Q10 Deficiency	*COQ8* c.811C > T 9(p.ARG)	18	72		No lesion		3.40	
25	Female	2,5	Leigh	*LINS gen* c.554a > G	12	12		Temporal		20	
26	Female	1,9	Leigh	*SURF1* c.530 T > G hom	12	12	3	No lesion		2.2	3 days
27	Male	6,9	Leigh	*MT-ND5* M.13513G > A	42	48		No lesion			
28	Female	18	Leigh	*SURF1 gene* c54 + 5G > T (IVS1 + 5G > T) hom	60	216	1	No lesion		28	
29	Female	1,4	POLG	c.3286C > T p.Arg1096h	12	15	1	No lesion	Bilateral central disorganization in sleep		
30	Male	7,3	POLG	c.1760C > T P.Pro587leu */c1760C > T.pro587leu	72	80	1	No lesion		36	
31	Female	9,1	POLG	*c.911 T > G* (p.Leu304Arg) hom	96	102	1	No lesion		12	
32	Female	12,5	FBXL4	*FBXL4* c.445G > A	1	11		No lesion		7.6	
33	Male	11	FBXL4	*FBXL4* c.445G > A	2	9		No lesion		10.2	
34	Male	18,5	LHON	*LHON* 11778G > A	156	156		No lesion		1.5	

Detailed patient-level data. MELAS, Mitochondrial encephalomyopathy, lacticacidosis, and stroke-like episodes syndrome; LHON, Leber’s hereditary optic neuropathy. n, frequency; %, percentage. For statistical tests, see the Methods section.

### Genetic diagnoses and distribution by diagnosis

Of the 34 patients with a genetic diagnosis of mitochondrial diseases, 44.1% (*n* = 15) were diagnosed with MELAS, 26.5% (*n* = 9) with Coenzyme Q10 deficiency, 11.8% (*n* = 4) with Leigh syndrome and 8.8% (*n* = 3) with *POLG* mutation. There were also cases with *FBXL4* mutation 5.9% (*n* = 2), LHON 2.9% (*n* = 1) (Table 1).

### Stroke-like episodes

**Figure 1:**
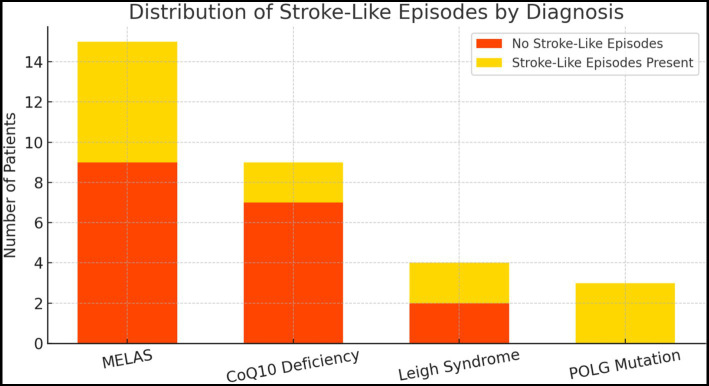
The graph shows the incidence of stroke-like attacks in individuals with different diagnostic groups. It is noteworthy that stroke-like attacks are more common in individuals with MELAS and POLG mutations. There is one patient diagnosed with ACATI and had a stroke attack. Abbreviations are explained in the figure legend. Red line indicates significance (*p* = 0.05).

Of the 13 patients with SLE, 46.1% (*n* = 6) were diagnosed with MELAS and 23.1% (*n* = 3) with POLG. These episodes were also observed in individuals diagnosed with Coenzyme Q10 deficiency 15.4% (*n* = 2), Leigh syndrome 15.4% (*n* = 2) (Figure 1). A comparative analysis of the data reveals that the most prevalent age group experiencing SLE was 5.01–10 years (38.4%), followed by 15.01–20 years (30.8%). The highest proportion of individuals without an episode was in the age group above 20 years, with 28.5%. However, no statistically significant relationship was found between age groups and SLE (*χ*^2^ = 6.703; *p* = 0.152) (Table 3).

**Table 3:** Age and stroke episode findings.

Age			Stroke-like attacks		Total	*χ*^2^	*p*
			Yes	No			
Age (years)	<5	n	3	4	7	6.703	0.152
		%	23.1%	19%	20.6%		
	5.01–10	n	5	3	8		
		%	38.4%	14.2%	23.5%		
	10.01–15	n	0	4	4		
		%	0.0%	19%	11.8%		
	15.01–20	n	4	4	8		
		%	30.8%	19%	23.5%		
	>20	n	1	6	7		
		%	7.7%	28.5%	20.6%		
Total		n	13	21	34		
		%	100.0%	100.0%	100.0%		

Chi-square test results are included (see *p* values).

### Clinical findings associated with stroke episodes

**Figure 2:**
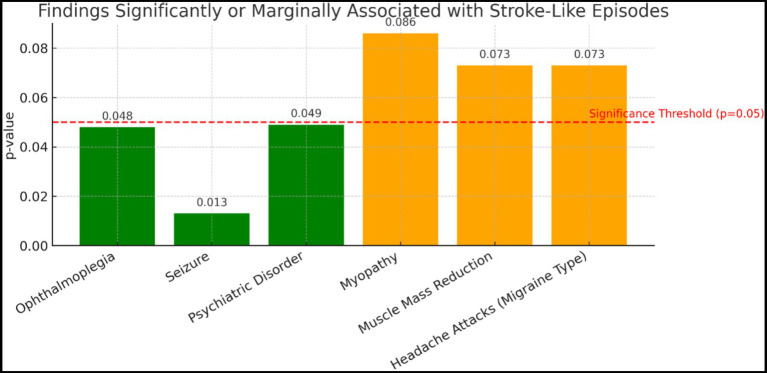
Distribution of stroke attacks and symptoms. In the graph, the red line indicates the significance limit (*p* = 0.05). Detailed distribution with statistical significance indicated.

The prevalence of seizures was found to be significantly higher in patients with a SLE compared to those without such an episode (64.3% vs. 22.7%, *p* < 0.05) (*p* = 0.013). Psychiatric disorders were also associated with SLE; being present in 44.4% of those who had an episode and 9.5% of those who did not (*p* = 0.049). Ophthalmoplegia was reported only in individuals who had an episode, and both findings were statistically significant (*p* = 0.048) (Figure 2).

When genetic subtypes were analyzed, migraine headache was reported 100% in *FBXL4* mutations, 88.9% in Coenzyme Q10 deficiency, 75% in Leigh syndrome, and 40% in MELAS. Cognitive decline was present in all patients (100%) with Leigh, *FBXL4* mutations and LHON. Decreased muscle mass was significantly observed in patients with MELAS, *FBXL4*. *C*ataracts were detected in 50% of individuals with *POLG* mutation only, which was distinctive in this respect. Hearing loss was observed in 50% of patients with *FBXL4* mutation. An evaluation of body mass index (BMI) revealed mean BMI values of 16.49 ± 3.85 in patients with MELAS, 23.83 ± 11.47 in patients with CoQ10 deficiency, 11.98 ± 2.97 in Leigh syndrome, and 27.34 ± 15.37 in the FBXL4, LHON group. Although no significant difference was found between the diagnosis groups (*χ*^2^ = 7.371; *p* = 0.061), the *p*-value is close to the significance limit and suggests that there may be BMI trends according to diagnosis.

### Seizures and EEG findings

A history of seizures was identified in 9/13 patients with stroke like episodes, while 4/13 had no history of seizures. In the evaluation of seizure types, 5/13 of patients experienced generalized seizures and 4/13 experienced focal seizures. Analysis of EEG findings revealed that EEG evaluation was not performed in 3/14 of the patients, whereas focal epileptiform activity was detected in 5/13. Additionally, generalized epileptiform activity was observed in 3/13 of patients, while 6/13 showed no evidence of generalized epileptiform activity (Table 4).

**Table 4:** Distribution of stroke patients (*n* = 13) regarding diagnosis and seizures.

Seizure and EEG finding		*n*	%/ (n/N)
Seizure	Yes	9	69.2 (9/13)
	No	4	30.8 (4/13)
Seizure Type	Focal seizure	4	30.8 (4/13)
	Generalized seizure	5	38.4 (5/13)
	No information	4	30.8 (4/13)
EEG Finding 1	No EEG	4	30.8 (4/13)
	Focal epileptiform activity	5	38.4 (5/13)
	No focal epileptiform activity	4	30.8 (5/13)
EEG Finding 2	Generalized epileptiform activity	3	23.1 (3/13)
	No generalized epileptiform activity	6	46.1 (6/13)
	No EEG	4	30.8 (5/13)
EEG Finding 3	Epileptiform activity in the left frontotemporal region	2	50 (2/4)
	Epileptiform activity in the temporaparietal region	1	25 (1/4)
	Epileptiform activity in the left hemisphere	1	25 (1/4)

Includes seizure type and EEG findings; values are given as *n* (%). n, frequency; %, percentage.

### Magnetic resonance imaging (MRI) findings

Cranial MRI revealed lesions in 15/30 of the patients. Of the lesions, 11/15 affected both hemispheres, 3/14 affected the left hemisphere, and 1/15 affected the right hemisphere. Lesions were most frequently observed in the frontal (4/11), temporal (4/11) and parietal lobes (3/11), with lower rates in the occipital lobe (1/11). Regarding lesion resolution, disappearance occurred within 2–6 months in 3/8 of patients, between 6 months and 1 year in 12.5% 1/8, and after more than 1 year in 4/8 of the patients with available data (Table 5).

**Table 5:** Cranial MRI findings.

Cranial MRI		*n*	% (n/N)
Cranial MRI	Lesion	15	51.7(15/30)
	No lesion	14	48.3 (14/30)
Localization Lesions 1	Right hemisphere	1	6.7 (1/15)
	Left hemisphere	3	20 (3/15)
	Bilateral hemispheres	11	73.3 (11/15)
Localization Lesions 2	Frontal	4	36.3 (4/11)
	Temporal	3	27.2 (3/11)
	Parietal	3	27.2 (3/11)
	Occipital	1	9 (1/11)
Time to Resolution of Lesions (Month)	2–6	3	37.5 (3/8)
	6–12	1	12.5 (1/8)
	>12	4	50 (4/8)
MR Spectroscopy	Not performed	21	61.8 (21/34)
	Lactate peak detected	5	38.5 (5/13)
	No lactate peak	8	61.5 (8/13)

n, frequency; %, percentage.

### MR spectroscopy findings

MR spectroscopy was performed in 13 / 34 of the patients; no lactate peak was found in 8/13, a lactate peak was found in 5/13 of the patients who underwent MR spectroscopy (Table 5).

### Laboratory findings

The mean plasma lactic acid level was 22.61 ± 23.24 mg/dL in individuals who had a SLE, while this value was 10.21 ± 12.39 mg/dL in those who did not have an episode. The difference between the two groups was not statistically significant (*z* = −1.785; *p* = 0.078).

### L-arginin treatment

A total of 12 patients (*n* = 10 MELAS, *n* = 1 Coenzyme Q10 deficiency, *n* = 1 Leigh syndrome) received L-arginine treatment. Among the 13 patients who experienced SLE, 6 received L-arginine therapy during the acute phase, and 4 of them continued long-term oral L-arginine treatment (Tables 2, 6).

**Table 6:** Stroke-like episodes and L-arginine treatment.

Stroke-like episodes			Arginine intake		Total	*χ* ^2^	*p*
			Present	Absent			
Stroke-like Episode	Present	n	6	7	13	1.087	0.297
		%	50.0%	31.8%	38.2%		
	Absent	n	6	15	21		
		%	50.0%	68.2%	61.8%		
Total		n	12	22	34		
		%	100.0%	100.0%	100.0%		

n, frequency; %, percentage.

## Discussion

In this multicenter observational cohort study, the clinical, radiological, EEG, and genetic features of genetically diagnosed mitochondrial diseases, the frequency of stroke-like episodes, associated findings, and L-arginine treatment were evaluated. In a study by Ng YS and colleagues on the prediction of stroke-like events and outcomes in mitochondrial diseases, 111 patients aged 1–72 years diagnosed with mitochondrial disease were retrospectively evaluated (13). This study indicated that stroke-like episodes in mitochondrial diseases are also seen in the paediatric age group. In our study, we evaluated paediatric patients who experienced stroke-like episodes associated with mitochondrial disease. Although the data obtained were generally consistent with the existing literature, they also showed some remarkable differences. SLE were most frequently observed in individuals diagnosed with MELAS (44.1%), as reported in the literature (11). In the majority of these patients (66.6%), m.3243A > G mutation was found in the *MT-TL1* gene, and this rate was consistent with previously reported data (14). *POLG* mutation stood out as the second most common cause of SLE, and this was consistent with the frequency ranking in the literature (13). Coenzyme Q10 (CoQ10) deficiency is one of the rare mitochondrial disorders that develop due to mutations in genes involved in ubiquinone synthesis. In the literature, there are a limited number of cases and small patient series in which SLE have been reported to be associated with mutations in biosynthesis genes such as COQ8A (ADCK3) and COQ4 (15, 16). In our study, SLE were observed in two of nine patients diagnosed with CoQ10 deficiency. This finding indicates that CoQ10 deficiency may rarely lead to SLE and emphasizes that neurologic symptoms should be carefully evaluated in this patient group. SLE associated with Leigh syndrome have rarely been described in the literature. In a case report, a three-month-old female infant with post-infectious encephalopathic features and neurologic symptoms, including hypotonia, apnea, and optic atrophy was diagnosed with Leigh syndrome. This case suggests that metabolic stress may trigger neurologic findings in Leigh syndrome, and the disease may present with stroke-like episodes (17). In our series, it was observed that two of the four patients with Leigh syndrome developed stroke-like episodes in relation to metabolic stress. This observation reveals that Leigh syndrome may present not only as a progressive neurodegenerative disorder but also with acute neurologic decompensations triggered by metabolic stress. It emphasizes the necessity for early diagnosis, close follow-up, and prevention of metabolic crises in this patient group. In a study conducted by Durrleman et al. (18) on 60 pediatric patients, it was reported that the first symptoms of the patients started between 0 and 28 months, and the first SLE occurred between 17 and 124 months. In a study conducted by Ng et al. (13) on Forecasting stroke-like episodes and outcomes in mitochondrial diseases, it was noted that 32% of patients experienced their first stroke episod after the age of 40. In our study, it was observed that the first symptoms usually started before the age of 5 years, and the first SLE occurred mostly in the age range of 5.01–10 years (38.4%). The youngest patient was 1.9 years old, and the oldest patient was 40 years old. This shows that the development of episodes is not limited by age and can occur at any age. Gender distribution was equal (F/M: 7/7), and gender was not a determining factor in episodes. In this respect, it differs from some studies in the literature (18). An analysis of the clinical symptoms revealed that headache was prevalent before a SLE. This was followed by decreased muscle mass, myopathy, and seizures. In a study by Xu et al. (14) involving children with MELAS syndrome, muscle weakness was defined as an indicator of severe mitochondrial dysfunction. In studies, seizures are frequently reported as the first symptom of SLE. In mitochondria-damaged cells, the increased metabolic demand associated with excessive neuronal activity during seizures cannot be met. This leads to local energy deficits, ion imbalances, and intracellular lactic acid accumulation. This situation can cause cell damage, vascular irregularities, and associated SLE. The high seizure frequency found in our study and its significant association with stroke- like episodes support the hypothesis that these episodes may be triggered by epileptic seizures (14, 18). In a study conducted by Ng et al. (13) on MELAS patients, it was reported that 91% of the patients had symptoms indicating mitochondrial dysfunction before the SLE, and the most common symptom was sensorineural hearing loss. In our series, findings such as hearing loss, cortical blindness, recurrent vomiting, and myopathy were frequently observed before SLE in individuals diagnosed with MELAS. In addition, sensorineural hearing loss was one of the most common symptoms in these patients. Although no statistical significance was found between SLE and some demographic variables such as age, gender, and BMI, low BMI in MELAS and Leigh patients is remarkable. This indicates that short stature and nutritional deficiency also accompany these patients and is in parallel with the literature (13). Studies have shown that brain lactate levels increase with disease progression in MELAS patients, and this increase reflects a progressive shift in oxidative redox potential. These findings suggest that lactate levels may be an important biomarker for monitoring the course of the disease and establishing a diagnosis (19). While studies suggested that lactate levels may be an important biomarker for monitoring the course of the disease and establishing a diagnosis, the variation does not clearly dichotamise those with or without stroke like episodes in our study. In radiologic evaluations, Durrleman et al. (18) reported that bilateral cortical diffusion hyperintensity and hyperperfusion were prominent in cranial MR images obtained in the first 48 h, and this condition developed due to energy metabolism disorders different from classical ischemic strokes (13). In our study, bilateral lesions were observed in all seven patients imaged in the acute period, and the most common site of involvement was frontotemporal lobes. When EEG findings were analyzed, focal epileptiform activity was found to be more frequent in accordance with the literature (13). Regarding the use of L-arginine treatment, a retrospective analysis by Ganetzky et al. (20) reported that clinical improvement was achieved in 47% of patients who received intravenous (iv) arginine treatment. A better response to treatment was observed, especially in cases of hemiplegic attacks. In our series, six patients with SLE received iv arginine treatment in the acute phase of the episode, and clinical improvement was observed. Seven of our patients who did not receive L-arginine treatment (3 POLG, 2 MELAS, 1 Coenzyme Q10 deficiency, 1 Leigh) experienced prolonged headaches, nausea and visual impairment. In Durrleman et al.’s (18) study, recurrent stroke-like episodes were reported in three patients over an eight-year follow-up period, whereas in our series, six patients experienced recurrent stroke-like episodes, with five episodes recorded in one MELAS patient and four in another. In systematic reviews on the efficacy of oral L-arginine treatment, it was emphasized that the clinical benefit of acute or prophylactic use was limited, but methodological deficiencies were found in these studies (21). In a study including six MELAS patients, it was reported that oral L-arginine treatment at a dose of 0.15–0.3 g/kg/day administered for 18 months resulted in significant improvement (22). In our study, 4 of our 13 patients with SLE continued long-term oral L-arginine treatment. Six of our MELAS patients without SLE also received long-term oral L-arginine treatment. While no episodes were observed in 6 of these patients, a median of 2 attacks were observed in 4 patients.

The study is robust in its examination of SLE in paediatric mitochondrial cases with molecular diagnosis, utilising multicentre and detailed clinical-imaging data. However, the small sample size, retrospective design, resulting lack of imaging data, and limited treatment outcomes represent significant limitations; conclusions regarding L-arginine efficacy are purely hypothesis-generating.

## Conclusion

This study showed that SLE in genetically diagnosed mitochondrial diseases are most commonly observed in MELAS and *POLG* mutations. However, they may also develop in rare subtypes such as CoQ10 deficiency and Leigh syndrome. During episodes, the presence of bilateral cortical lesions on cranial MRI—often located in the frontotemporal regions and inconsistent with vascular distribution—and the detection of focal epileptiform activity on EEG were diagnostically significant findings. Clinical improvement was observed with iv L-arginine treatment initiated in the acute period. The findings emphasize that Stroke-like episodes should be considered in the differential diagnosis of sudden onset neurological findings in mitochondrial diseases.

## Data Availability

The datasets presented in this study can be found in online repositories. The names of the repository/repositories and accession number(s) can be found in the article/supplementary material.
